# Validation of the Pelvic Organ Prolapse Simple Screening Inventory (POPSSI) in a population of Ethiopian women

**DOI:** 10.1186/s12905-019-0746-x

**Published:** 2019-04-03

**Authors:** Dawit Worku Kassa, Yirgu Gebrehiwot Ferede, Polina Advolodkina

**Affiliations:** 1 Addis Ababa University School of Medicine, Addis Ababa, Ethiopia; 2 Emory university school of medicine, Atlanta, USA

**Keywords:** POP, POPSSI, Ethiopian women

## Abstract

**Background:**

The incidence of Pelvic Organ Prolapse (POP) in the developing world is not known. A nonclinical screening tool for prolapse is needed in the resource poor setting. In this study, we aim to determine the validity of the Pelvic Organ Prolapse Simple Screening Inventory (POPSSI) for detection of POP in a population of women at two academic hospitals in Addis Ababa, Ethiopia.

**Methods:**

Women from two teaching hospitals in Addis Ababa, Ethiopia were recruited to complete the POPSSI questionnaire as well as a Pelvic Organ Prolapse Quantification (POP-Q) exam. Descriptive data on exam findings were collected. Questionnaire responses were then correlated to exam findings and data analyzed to determine the validity of this test as a screening tool for prolapse in our patient population.

**Results:**

Majority of the women with POP had advanced stage. The sensitivity and specificity of the POPSSI for identifying pelvic organ prolapse in our study patient population was 91.7 and 60.6% respectively.

**Conclusion:**

The POPSSI has a high sensitivity for detecting women with POP. “Feeling or seeing bulge” had a higher sensitivity and specificity.

## Background

Pelvic Organ Prolapse (POP) is estimated to affect 50% of parous women in the developed world and about 2–3% of nulliparous women below the age of 60 [1–3]. POP results in significant morbidity and likewise, is associated with high health-care burden [4]. POP results from the failure of the pelvic floor to support one or more visceral organs. The resultant descent of pelvic organs can lead to pressure symptoms, defecatory and urinary dysfunction, sexual dysfunction, and considerable impact on quality of life. In the developing-world, several small community-based studies have demonstrated significant morbidities attributable to POP with effects on patient’s emotional well-being, interpersonal relationships, and ability to work [5–7]. POP is challenging to study in a resource poor setting because accurate diagnosis of POP requires access to skilled medical care. This represents a significant gap in medical knowledge because women in resource poor settings may be uniquely exposed to factors that predispose to pelvic floor disorders. Specifically, women in much of the developing world have higher fertility rates, younger age at first pregnancy, less reliable access to obstetric care, and higher rates of malnutrition as compared to the developed world. The Pelvic Organ Prolapse Quantification System (POP-Q) is a reproducible, site-specific, and quantitative system for staging pelvic support which has been broadly used in the medical literature to describe prolapse. In a resource-poor community setting, the POP-Q exam is not feasible due to limitations in access to care. Several screening questionnaires for POP have been proposed and the best studied has been the Pelvic Floor Distress Index (PFDI). However, this questionnaire is long and cumbersome and therefore limited in feasibility in the resource poor setting. In this paper, we validate the Pelvic Organ Prolapse Simplified Screening Inventory (POPSSI) which was adapted from the Pelvic Floor Disorder Inventory (PFDI). While the PFDI is a detailed questionnaire consisting of 20 questions focusing on prolapse, anorectal, and urinary symptomatology, the POPSSI consists of only 4 questions: 1) Do you experience urinary incontinence with laughing, sneezing or coughing? 2) Do you experience urinary urgency? 3) Do you feel pain during defecation? 4) Do you feel or see a bulge in the vagina? The POPSSI was originally studied in an Iranian patient population and the original authors of that paper reported an 87.4% specificity for detection of POPSSI in asymptomatic women and a 96.7% sensitivity among women with subjective POP complaints [8]. Our study population consists of women seeking gynecologic care at two tertiary teaching hospitals in Addis Ababa, Ethiopia. The women in this group come from diverse regions around Ethiopia and represent a broad sample of the Ethiopian population. Ethiopian women have unique epidemiological characteristics which may predispose to pelvic floor disorders including high rates of malnutrition, early age at first marriage and childbearing, high fertility rates, and very poor access to obstetric care. Specifically, high fecundity and early age at first marriage may contribute to a high prevalence in this population. In a 2016 Demographic and Health Survey, Ethiopian women were reported to be married at a median age of 17. The total fertility rate among Ethiopian women is higher than in the developed world with 4.6 children born per woman in 2016. Teenage pregnancy has been steadily decreasing since 2000, but still represents a sizable proportion of the population, with 13% of women age 15–19 having begun childbearing. Finally, Ethiopian women generally have limited access to obstetric care and are therefore at an increased risk of obstetric-related pelvic floor injury with 95% of all births occurring at home prior to 2000 [9]. All of these factors make the women of Ethiopia an ideal patient population to study in the validation of the POPSSI as a low-cost and culturally feasible screening tool.

## Methods

Our study population consists of women who sought gynecologic care at Ghandi Memorial and Zewditu Memorial Hospital in the time period of May to August of 2014. Ghandi and Zewditu Memorial represent two tertiary care centers in central Addis Ababa, Ethiopia. The study was approved by each hospital’s ethical review board. A written letter of approval was obtained from the original publishers of the POPSSI study for use of the questionnaire. Women aged 45 years or older were considered eligible for study participation and all women who met inclusion criteria were offered to participate. Exclusion criteria included inability or unwillingness to give consent, inability to understand spoken Ethiopian Amharic, and any contraindication to vaginal examination. Women who presented for a chief complaint of prolapse were excluded. The main reason why women who were already diagnosed with POP or on treatment for POP were excluded from the study is that we wanted to validate and later use the tool for screening women who may not present complaining prolapse. Women that comes to the gynecologic outpatient clinic who consented for the study and fulfilled the inclusion criteria were asked the four questionnaires and followed by physical examination using the POP-Q system. Those with at least stage one prolapse then became the study and those without POP became the control group. Basic demographic data on patient Age and parity was collected. The POPSSI questionnaire was translated into Ethiopian Amharic using a translation-back-translation method as follows: The English version of the POPSSI was translated to Amharic language by three independent individuals fluent in Amharic, English and medical terminology. Three other independent translators then translated the Amharic version back to three English versions for each questionnaire. The newly created English version was then compared to the original English version to ensure comparability of the language and similarity of interpretation by two individuals fluent in English and medical terminologies. A scoring system was utilized to determine which of the three Amharic translations would be used. Two investigators compared each English back translated version with an item- by – item rating using the Likert scale. Given that a substantial proportion of our study population is illiterate, a research assistant was utilized to read consents and the POPSSI questionnaires to patients and to collect response data. Patient’s responses to the four POPSSI questions were scored as “yes” or “no” with “no” responses being assigned a value of 0. In addition to the questionnaire, each patient in the study had a standard pelvic exam with a skilled and experienced gynecologist. Data was collected on clinical stage of pelvic organ prolapse as well as specific findings of the lead-point of the prolapse. All data were then used to calculate sensitivity, specificity, likelihood ratios using SPSS version 20.0 statistical software. The Receiver Operator Characteristics (ROC) was calculated for the study questionnaire.

## Results

**Fig. 1:**
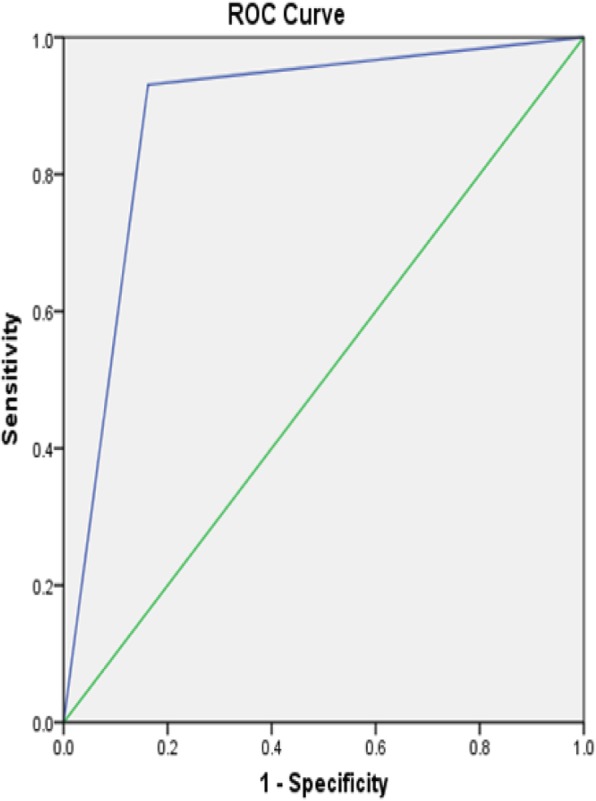
Receiver operating characteristics curve for using “feeling or seeing of bulge in the vagina” to predict anterior compartment prolapse

**Fig. 2:**
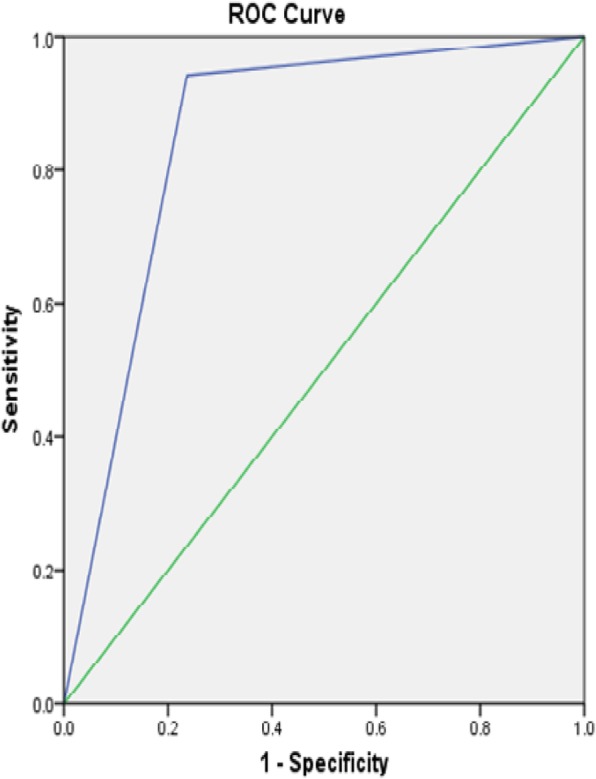
Receiver operating characteristics curve for using “feeling or seeing of bulge in the vagina” to predict an apical prolapse

**Fig. 3:**
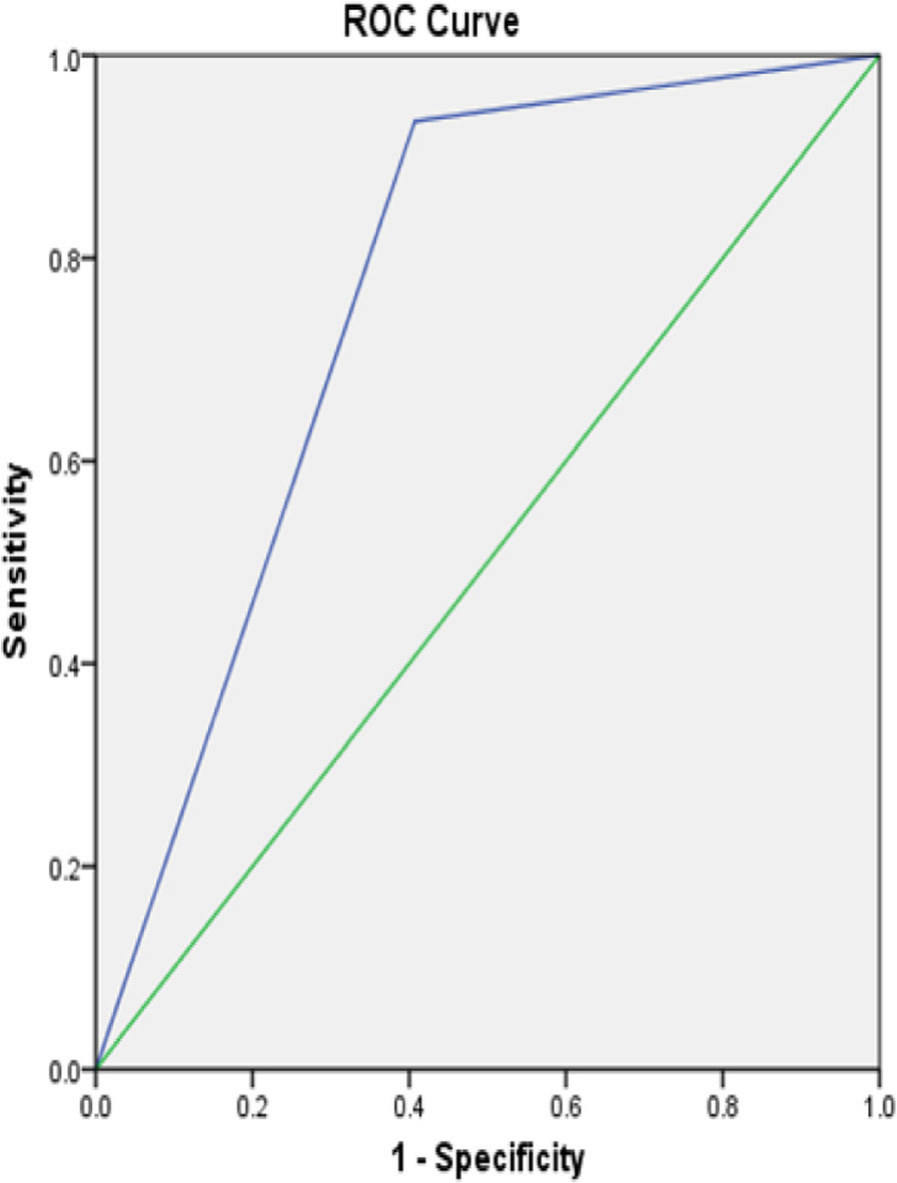
Receiver operating characteristics curve for using “feeling or seeing of bulge in the vagina” to predict posterior compartment prolapse

**Fig. 4:**
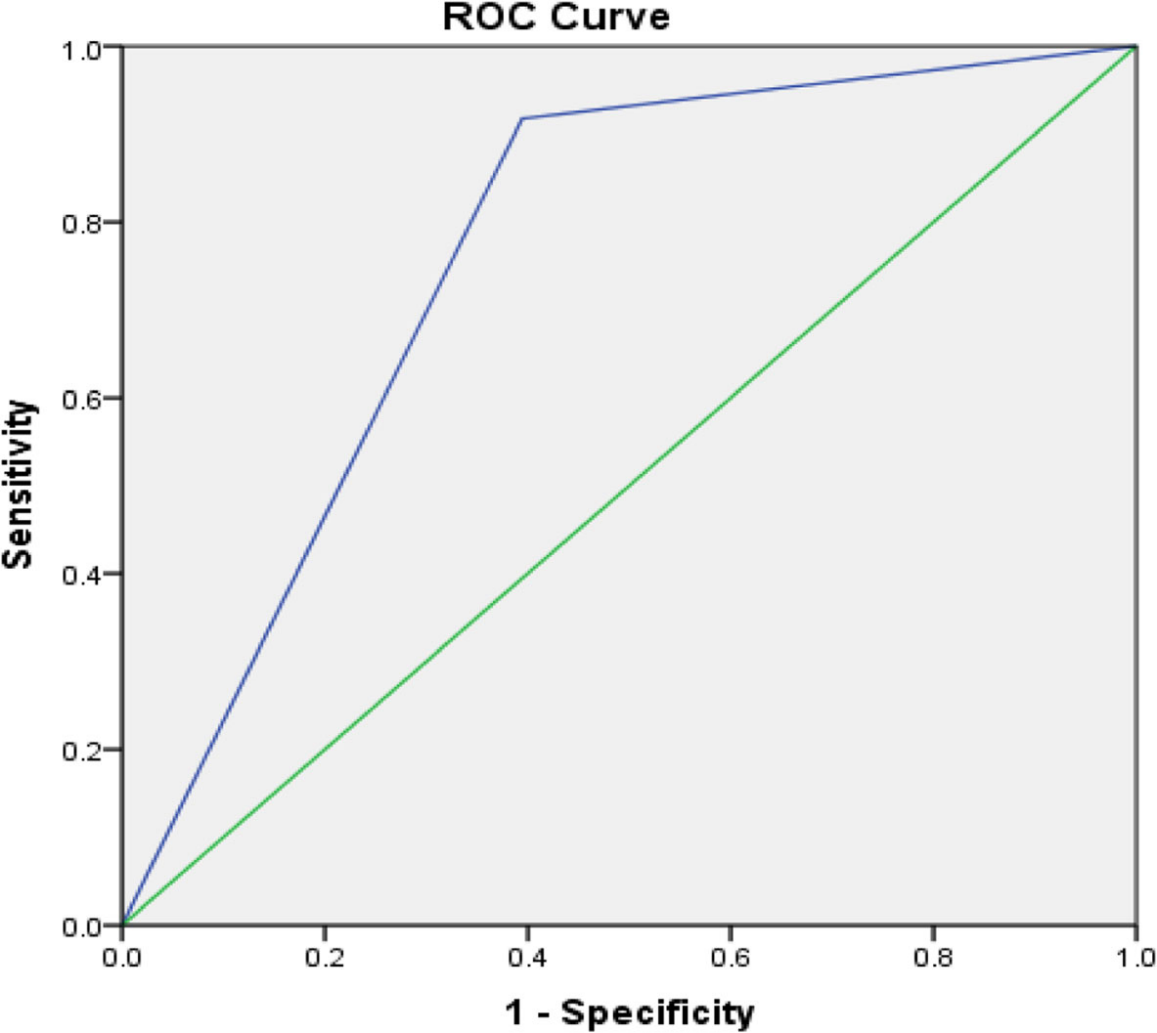
Receiver operating characteristics curve for POPSSI to predict POP

One hundred and nine patients meeting the above criteria were classified as having pelvic organ prolapse during the study period. Likewise, 109 patients were concurrently identified with no clinically evident prolapse and served as controls in this study. The mean age of all participants was 49.9 years. Age did not differ greatly between the prolapse and reference groups (49.9 ± 11 and 49.9 ± 9.2 respectively). Women with prolapse had higher parity, median parity of 4, as compared with those without prolapse, median parity of 3. Of the women classified as having prolapse, 46.8% had anterior compartment prolapse with an apical defect and Majority of the women in the prolapse group had Stage IV disease [Tables 1, 2, and 3,]. Unfortunately, the final response rate from all women invited to participate is not known. We then analyzed each component questions of the POPSSI independently for its utility in detecting POP. Self-reported stress urinary incontinence (leakage of urine following laughing, sneezing, or coughing) had a very low sensitivity for detecting POP, ranging from 16.8 to 28.3%. As with urinary incontinence, pain with defecation also had a low sensitivity for prolapse that ranged from 7.9 to 15.2%. The sensitivity and specificity of urinary urgency was 39.6 and 65.8% for the detection of anterior compartment prolapse, 37 and 63.4% for posterior compartment prolapse, 46 and 69.5% for apical prolapse respectively. “Feeling or seeing bulge in the vagina” had high sensitivity and specificity for detecting anterior compartment prolapse, posterior compartment prolapse and an apical prolapse [Table 3]. The sensation of a bulge in the vagina had a sensitivity and specificity 93.1 and 83.8% respectively with +LR of 5.47 and –LR of 0.08 for anterior compartment prolapse with a maximum sensitivity of 98% for stage IV disease. The ROC analysis showed 0.884 [Fig. 1]. The sensitivity and specificity of presence of a bulge in the vagina for the detection of an apical prolapse were 94.3 and 76.3% respectively with +LR 3.91, −LR 0.07 and the maximum sensitivity, 96.8%, was for stage IV apical prolapse. The ROC analysis showed the area under the curve value of 0.853 [Fig. 2]. Finally, the sensitivity and specificity of presence of feeling or seeing bulge in the vagina for the detection of posterior compartment prolapse were 93.5 and 59.3% respectively with +LR 2.26, −LR 0.11 and the maximum sensitivity of 100% was for stage IV disease. The ROC analysis showed the area under the curve value of 0.764 [Fig. 3]. In this study, the overall sensitivity and specificity of the POPSSI in detecting women with pelvic organ prolapse was 91.7 and 60.6% respectively and the calculated area under the ROC was 0.761 [Fig. 4].

**Table 1:** Frequency distribution of the type of POP

Type of prolapse	Frequency	%
Isolated anterior compartment prolapse	12	11
Isolated posterior compartment prolapse	4	3.7
anterior compartment prolapse with posterior compartment prolapse	6	5.5
anterior compartment prolapse with apical prolapse	51	46.8
Apical prolapse with posterior compartment prolapse	4	3.7
All the three types	32	29.4
Total	109	100

**Table 2:** Frequency distribution of the stage of POP

POP	Stage 0 (No disease)	Stage 1	Stage 2	Stage 3	Stage 4	Total
Anterior compartment prolapse	8 (7.3%)	3 (2.8%)	16 (14.7%)	33 (30.3%)	49 (45%)	109 (100%)
Posterior compartment prolapse	63 (57.8%)	0	12 (11%)	16 (14.7%)	18 (16.5%)	109 (100%)
Apical prolapse	22 (20.2%)	0	8 (7.3%)	17 (15.6%)	62 (56.8%)	109 (100%)

**Table 3:** Sensitivity, specificity, PPV and NPV of POPSSI for detecting POP

POPSSI	anterior compartment prolapse				posterior compartment prolapse				Apical prolapse			
	Sensitivity (%)	Specificity (%)	PPV (%)	NPV (%)	Sensitivity (%)	Specificity (%)	PPV (%)	NPV (%)	Sensitivity (%)	Specificity (%)	PPV (%)	NPV (%)
SUI	16.8	82.1	44.7	53.3	28.3	85.5	34.2	81.6	16.1	81.7	63.1	59.4
Urinary Urgency	39.6	65.8	50	55.7	37	63.4	21.2	78.9	46	69.5	50	65.9
Pain during defecation	7.9	95.7	61.5	54.6	15.2	96.5	53.8	80.9	5.7	93.5	38.4	60
Feeling or seeing bulge	93.1	83.8	83.1	93.3	93.5	59.3	38	97.1	94.3	76.3	72.5	95.2

## Discussion

Although several screening tools have been introduced to identify women suffering from POP, there is no consensus on a simple and accurate tool. This represents a substantial gap in medical knowledge, especially in our understanding of POP among women in the developing world. In our study, we validate a simple screening tool known as the POPSSI which was modified from the PFDI for the detection of POP among Ethiopian women. The original study validating the POPSSI was done among Iranian women. Here, we have demonstrated several key differences as compared to the Iranian study. In contrast to the Iranian study, our study found that stress urinary incontinence (having urinary incontinence with laughing, sneezing or coughing) had a much lower sensitivity for POP of 16.8% as compared to the Iranian study of 70.4%. This could be due to the fact that majority of women with POP presented with an advanced disease. Nevertheless, we found that urinary urgency and pain with defecation had higher sensitivities for POP (39.6 and 7.9% respectively) as compared to the Iranian study (21.3 and 3.9%) with high specificities.

In the original validation study of the POPSSI questionnaire, the authors of the study reported an overall sensitivity and specificity of 45.5 and 87.4% of POPSSI for the detection of POP among a general population of Iranian women [4]. In our study population of women seeking gynecologic care at two tertiary care centers in Addis Ababa, Ethiopia, we found that the sensitivity and specificity of POPSSI for identification of pelvic organ prolapse was 91.7 and 60.6% respectively. These higher values could be the reflection of higher proportion of women in our study presenting with advanced stage (Stage IV) POP. And of the four questions of the POPSSI, “feeling or seeing bulge” had a higher sensitivity and specificity for detecting POP. A potential limitation of our study is that we have elected to study women who sought care in tertiary care setting and therefore were likely to have severe gynecologic diseases and the discrepancy that was seen from the Iranian study could be mainly due to this fact. We have attempted to minimize selection bias by eliminating those women who sought care specifically for POP from the study.

## Conclusion

Overall, our study shows a potential for the POPSSI questionnaire as a screening tool for POP. The POPSSI can detect POP in 90% of affected gynecologic patients seen in our clinical setting with 60% accuracy. A future community-based study could attempt to validate and use the POPSSI in a general population in Ethiopia.

## Abbreviations

- LR: Negative likelihood ratio
+LR: Positive likelihood ratio
NPV: Negative predictive value
PFDI: Pelvic floor disorder inventory
POP: Pelvic organ prolapse
POP-Q: Pelvic organ prolapse quantification
POPSSI: Pelvic organ prolapse simple screening inventory
PPV: Positive predictive value
ROC: Receiver Operator Curve
